# Computational Detection of Known Pathogenic Gene Fusions in a Normal Tissue Database and Implications for Genetic Disease Research

**DOI:** 10.3389/fgene.2020.00173

**Published:** 2020-02-28

**Authors:** Gavin Robert Oliver, Garrett Jenkinson, Eric W. Klee

**Affiliations:** ^1^Center for Individualized Medicine, Mayo Clinic, Rochester, MN, United States; ^2^Department of Health Sciences Research, Mayo Clinic, Rochester, MN, United States

**Keywords:** fusion transcript, RNA-Seq, rare genetic disease, normal tissue, GTEx

## Abstract

Several recent studies have demonstrated the utility of RNA-Seq in the diagnosis of rare inherited disease. Diagnostic rates 35% higher than those previously achievable with DNA-Seq alone have been attained. These studies have primarily profiled gene expression and splicing defects, however, some have also shown that fusion transcripts are diagnostic or phenotypically relevant in patients with constitutional disorders. Fusion transcripts have traditionally been studied as oncogenic phenomena, with relevance only to cancer testing. Consequently, fusion detection algorithms were biased toward the detection of well-known oncogenic fusions, hindering their application to rare Mendelian genetic disease studies. A recent methodology published by the authors successfully tailored a traditional algorithm to the detection of pathogenic fusion events in inherited disease. A key mechanism of decreasing false positive or biologically benign events was comparison to a database of events detected in normal tissues. This approach is akin to population frequency-based filtering of genetic variants. It is predicated on the idea that pathogenic fusion transcripts are absent from normal tissue. We report on an analysis of RNA-Seq data from the genotype-tissue expression (GTEx) project in which known pathogenic fusions are computationally detected at low levels in normal tissues unassociated with the disease phenotype. Examples include archetypal cancer fusion transcripts, as well as fusions responsible for rare inherited disease. We consider potential explanations for the detectability of such transcripts and discuss the bearing such results have on the future profiling of genetic disease patients for pathogenic gene fusions.

## RNA Sequencing in Rare Disease

The study of rare inherited disease has been a major beneficiary of the next-generation sequencing era. Following the first reports of diagnoses arising from exome (Choi et al., 2009; Ng et al., 2009) and genome sequencing (Lupski et al., 2010), the number of success stories has risen as studies have increased in size and number. Cohort-based studies have reported diagnostic rates of 18–40% (Yang et al., 2013; Posey et al., 2016; Sawyer et al., 2016) and for several years numbers in this range came to represent a *status quo* in the field. A 2017 paper utilizing RNA-Seq (Cummings et al., 2017) presented a forward stride in diagnostic yield by reporting a 35% improvement over DNA-Seq alone, in a study of muscular pathologies. Almost simultaneously, a second paper focused on mitochondriopathies (Kremer et al., 2017) employed similar RNA-Seq analyses to attain an increase in diagnostic yield of 10%, while a third paper (Fresard et al., 2019) reported a diagnostic yield increase of 7.5% in a study of phenotypically diverse individuals. Collectively these studies reported on RNA-based abnormalities in gene expression levels, splicing patterns and allelic imbalances. In parallel to these landmark publications, the authors of this perspective published a series of case studies and research articles (Cousin et al., 2018; Oliver et al., 2019a, b) highlighting the diagnostic utility of fusion transcript profiling in studies of rare, undiagnosed disease. These publications report on the diagnosis of severe combined immunodeficiency (diagnosed by reciprocal *ATM-SLC35F2* fusion), and an instance of multiple exostoses (diagnosed by *SAMD12-EXT1* fusion), as well as five additional experimentally validated fusion transcripts with potential phenotypic relevance. In this cohort of undiagnosed patients with diverse phenotypes, a total diagnostic improvement of 4.3% was attained. The cases diagnosed through fusion detection had escaped diagnosis with a broad assortment of clinical and research assays, including methods specifically targeting the genes later determined to be disrupted by the identified fusion transcripts. We concluded that fusion transcript detection should be a core component of any RNA-Seq analysis aimed at diagnosis of rare disease and that genes previously dismissed as unimpaired by gold-standard clinical testing could in fact be revealed as functionally abrogated utilizing such RNA-based analysis.

## Adapting Fusion Detection to Rare Disease

**FIGURE 1:**
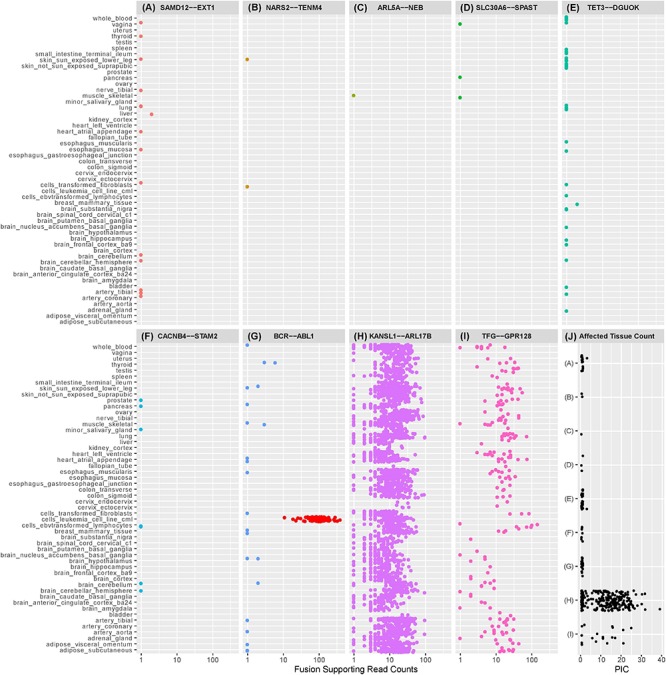
Dot plots illustrating the number of observations of selected exon-exon fusion transcripts in the GTEx RNA-Seq data by tissue type. Fusion analysis was performed using RNA-Seq data from 8187 samples passing QC, representing 549 individuals and 52 tissue types, extracted from GTEx (version 6p). Fusion transcript identification was performed using STAR-Fusion (Haas et al., 2017) with default settings following STAR (v2.5.2b) two-pass alignment (Dobin et al., 2013). Similar to our previously described methods, preliminary fusion calls were used to maximize sensitivity by avoiding default filters encoded in the callers (Oliver et al., 2019b). Fusion-supporting junction and spanning reads identified by STAR Fusion were combined into a single supporting read count for each event. Fusions **(A)–(F)** are fusion candidates originating from a cohort analysis of rare disease patients previously published by the authors (Oliver et al., 2019b). Five fusions experimentally validated in the authors’ cohort analysis were not observed in the GTEx database and are not displayed in the figure. *SAMD12-EXT1* **(A)** was detected in the authors’ cohort study and demonstrated to be a pathogenic event responsible for the rare condition of multiple exostoses. Candidate *SAMD12-EXT1* fusions sharing the same exon-exon boundaries were later shown to be detectable with limited read support in a subset of tissues for five healthy individuals in GTEx. A selection of alternative exon-exon *SAMD12-EXT1* fusions were observed in 10 further healthy individuals. The oncogenic *BCR-ABL1* **(G)** was detectable in 22 healthy individuals, although with limited read support and within a small subset of tissues. Limited read support observed in healthy individuals contrasts strongly with the substantial read support visible in leukemia cell lines (red dots). *KANSL1-ARL17B* **(H)** and *TFG-GPR128* **(I)** are previously described polymorphic fusion events, observed here in larger numbers of patients and tissues, with greater read support than the pathogenic or suspected pathogenic fusions originating from the authors’ cohort study. **(J)** shows the per-individual affected tissue count (PIC) for each healthy individual for which a fusion candidate was detectable. Each dot represents the number of tissues containing the relevant fusion in a single individual. Fusions in **(J)** are labeled **(A–I)** corresponding to the fusions appearing in plots **(A–I)**. Pathogenic or potentially pathogenic fusions from the authors’ cohort study are detectable in small numbers of tissues per individual, similarly to the known pathogenic *BCR-ABL1* fusion event. Polymorphic fusions are detectable in larger numbers of tissues per healthy individual.

Pathogenic fusion transcript detection in inherited disease is particularly notable as it has been traditionally associated with oncology. Initially believed to be isolated to blood-based neoplasia (Daley and Ben-Neriah, 1991) and later shown to be common in solid tumors (Barr, 1998; Aman, 1999), fusion transcripts received significant attention due to their diagnostic, prognostic and sometimes remarkable therapeutic implications (Burchill, 2003; Schnittger et al., 2003; An et al., 2010). Discussion of fusion transcripts detected in normal tissues centered on apparently benign events resulting from co-transcription of neighboring genes or more controversially from trans-splicing (Akiva et al., 2006; Peng et al., 2015; Babiceanu et al., 2016; Yuan et al., 2017; He et al., 2018). Reports of fusions in the context of inherited disease existed only in isolated case studies and were not systematically reported on until 2019 (Oliver et al., 2019b). The formulation of computational fusion detection software reflected the field’s focus on oncology-related fusion events and algorithms were primarily trained using incompletely characterized tumors or cancer cell-lines (Kumar et al., 2016). Algorithm performance was known to falter when analyzing data types or tissue sources distinct from their training data due to overfitting of filtering criteria (Kumar et al., 2016) and consequently these methods may have been expected to perform sub-optimally when newly applied to the study of rare germline disease. A further possible confounding factor is that well-characterized oncogenic fusions are protein-coding, gain-of-function events with relatively abundant RNA expression. Conversely, rare genetic diseases are frequently caused by loss-of-function events, where RNA may be subject to nonsense mediated decay, and causal fusions are likely to have relatively low RNA expression. Thus, detection algorithms primarily trained with oncogenic fusions may be biased by these and not optimized to account for different expression levels and patterns of read support. Such difficulties were demonstrated in our study where TopHat Fusion (Kim and Salzberg, 2011) using default parameters succeeded in detecting only one of eight fusion events detected and laboratory-validated in our rare disease cohort (Oliver et al., 2019b). To address this, we implemented a series of filtering and classification steps to detect fusions potentially linked to rare genetic constitutive disease. A core component of this strategy was a database of candidate fusion transcripts computationally detected in healthy tissue. The rationale was similar to filtering strategies using variant population frequencies from databases like gnomAD or ExAC (Lek et al., 2016) to exclude common variation when seeking the cause of rare genetic disease. By performing fusion analysis on 8,187 RNA samples representing 549 individuals and 52 tissue-types from the gene tissue expression (GTEx) database (Carithers et al., 2015) we created a database of fusion events detectable in healthy tissue (see Figure 1 legend for methodology). Using this resource, recurrent events arising from immunoglobulin rearrangements, unannotated transcripts, and read-through transcription could be annotated and deprioritized from further interpretation. Similarly, recurrent artifacts arising from analytical errors such as misalignments or laboratory protocol artifacts could be tagged and filtered, avoiding further consideration. Since GTEx consists of healthy tissues donated by individuals free from early onset inherited disease (post-mortem), the potential for them carrying events causal of rare undiagnosed disease, while possible (e.g., an incompletely penetrant event or a single, recessive event) could be estimated to be very low in a database containing tissue from 549 donors. Furthermore, a pathogenic transcriptomic phenomenon traditionally believed to be isolated to cancer (Aman, 1999, 2005) and only recently attributed to the causation of rare disease, could reasonably be predicted to be wholly absent from normal tissues. Based on these hypotheses, a simple exclusionary filter stating *if fusion candidate A is observed in the normal tissue database, filter fusion candidate A from the putative causal list for a diseased individual* would seem logical. However, a more complicated reality became evident when we evaluated the fusion data from our analysis of the GTEx database.

## Pathogenic Fusions in Normal Tissues

Our GTEx fusion database was queried for exon to exon fusions involving the gene pairs comprising eleven fusion candidates reported in our prior study (Oliver et al., 2019b; Table 1 rows 1–10). Three of the fusions are classified as known pathogenic events while eight are classified as potentially pathogenic since they involve genes linked to the patient phenotypes. Eight of the eleven fusion products were previously validated in our study by orthogonal technologies (Table 1), including the aforementioned pathogenic loss-of-function events affecting genes strongly linked to the patients’ phenotype (reciprocal *ATM-SLC35F2* and *SAMD12-EXT1*). We specifically profiled the GTEx database for exon to exon fusions as these were believed likely to be most technically robust. The rationale underlying this assertion is that spurious artifactual events are unlikely to generate fusions at precise exon-exon boundaries but rather offer increased confidence that a splicing-related mechanism has given rise to the transcript species and they are therefore likely true biological events. Conversely, candidate fusions between two genes that involve random intra-exonic or intronic sequence have higher potential of representing artifactual data (although not every case will be an artifact).

**TABLE 1:** Fusion candidates assessed for presence in the GTEx normal tissue fusion database.

**Fusion**	**Previously validated**	**Biological relevance**	**Present in GTEx?**	**Source**
*ATM-SLC35F2* and *SLC35F2-ATM*	Yes – ddPCR and PCR of RNA, sequencing of DNA	Causative of severe combined immunodeficiency	No	Cousin et al., 2018 and Oliver et al., 2019b
*SAMD12-EXT1*	Yes – ddPCR and PCR of RNA, aCGH and molecular inversion probe analysis of DNA	Causative of multiple exostoses	Yes	Oliver et al., 2019a, b
*NARS2-TENM4*	Yes – ddPCR and PCR of RNA	Potentially pathogenic	Yes	Oliver et al., 2019b
*C18orf32-DYM*	Yes – ddPCR of RNA	Potentially pathogenic	No	Oliver et al., 2019b
*ARL5A-NEB*	Yes – ddPCR of RNA	Potentially pathogenic	Yes	Oliver et al., 2019b
*SON-FCRL3*	Yes – ddPCR of RNA	Potentially pathogenic	No	Oliver et al., 2019b
*PDPK1-PRSS21*	Yes – ddPCR and PCR of RNA, aCGH of DNA	Potentially pathogenic	No	Oliver et al., 2019a, b
*SLC30A6-SPAST*	No – negative ddPCR and PCR of RNA	Potentially pathogenic	Yes	Oliver et al., 2019b
*TET3-DGUOK*	No – negative ddPCR and PCR of RNA	Potentially pathogenic	Yes	Oliver et al., 2019b
*CACNB4-STAM2*	No – negative ddPCR and PCR of RNA	Potentially pathogenic	Yes	Oliver et al., 2019b
*BCR-ABL1*	Yes – extensively published	Oncogenic in several leukemias	Yes	Daley and Ben-Neriah, 1991 and others
*TMPRSS2-ERG*	Yes – extensively published	Oncogenic primarily in prostate cancer	No	Tomlins et al., 2008 and others
*FRFR2-TACC3*	Yes – extensively published	Oncogenic in cholangiocarcinoma and other solid tumors	No	Costa et al., 2016 and others
*ALK-EML4*	Yes – extensively published	Oncogenic primarily in lung cancer	No	Sabir et al., 2017 and others
*SLC45A3-ELK*	Yes – extensively published	Oncogenic primarily in prostate cancer	No	Rickman et al., 2009 and others
*KANSL1-ARL17B*	Yes – extensively published	Polymorphic	Yes	Boettger et al., 2012 and others
*TFG-GPR128*	Yes – extensively published	Polymorphic	Yes	Chase et al., 2009 and others

*Eleven candidates (rows 1–10) originated in prior studies published by the authors. Eight of these were previously experimentally validated and three (one reciprocal) were confirmed pathogenic while the remainder were classified as potentially pathogenic as they involve genes linked to the patient phenotypes. Rows 11–15 describe known pathogenic fusions previously published extensively by others. Rows 16–17 describe known polymorphic events previously published by others.*

Five of the eleven fusion gene pairs showed no evidence of exon-exon fusions within the GTEx database. All five fusions not detected in GTEx had been experimentally validated in our prior study (Table 1) and included the pathogenic reciprocal *ATM-SLC35F2* event. The remaining six fusion gene pairs appeared in the GTEx fusion database (Figure 1) and included three which were experimentally validated in our prior study. No obvious differences were observed between previously validated (Figures 1A–C) and unvalidated events (Figures 1D–F), in terms of the number of tissues or patients in which they were observed. Surprisingly, the pathogenic *SAMD12-EXT1* fusion was present in five independent patient samples in the GTEx database (Figure 1A), and fused at the same exon boundaries observed in our study. It was considered possible that individuals with bone exostoses might have been included in the GTEx cohort, however, the fusion was only observed in transformed fibroblasts (one individual), esophageal mucosa (one individual), sun-exposed skin of the lower leg (one individual) and lung tissue (two individuals). Notably these observed fusions occurred in a limited number of tissues (maximum one per individual) and with limited read-support (only a single supporting read per patient). *SAMD12-EXT1* fusions with other boundaries were identified in an additional 10 individuals with one individual showing evidence of three distinct *SAMD12-EXT1* candidates joined at different exon boundaries in three different tissues.

The presence of the pathogenic *SAMD12-EXT1* fusion in normal tissues led us to question if other pathogenic fusion events might be detectable in normal tissues. We selected pathogenic fusions including *BCR-ABL1, TMPRSS2-ERG, FRFR2-TACC3, ALK-EML4*, and *SLC45A3-ELK* from the literature (Table 1 rows 11–15; Daley and Ben-Neriah, 1991; Tomlins et al., 2008; Rickman et al., 2009; Costa et al., 2016; Sabir et al., 2017). Of these, *BCR-ABL1* which is arguably the archetypal gene fusion (Table 1 row 11 and Figure 1G) and the first pathogenic gene fusion to be described (Parker and Zhang, 2013) was also observed in the GTEx cohort. This fusion is an oncogenic driver in several forms of leukemia and a well-studied and successful drug-target (An et al., 2010). The classical *BCR* exon 14 to *ABL1* exon 2 fusion was computationally detectable in 22 patients (Figure 1J) with a very similar technical profile to *SAMD12-EXT1* (i.e., only one tissue per patient, generally only one to two supporting reads per event and generally occurring in tissue unrelated to its known oncogenic environment). For purposes of comparison, we evaluated lymphoma cell lines in the GTEx database and observed starkly different levels of read support for the *BCR-ABL1* fusion. While number of fusion-supporting reads in healthy tissues was typically less than two, the cell lines contained tens to hundreds of supporting reads (Figure 1G).

## Polymorphic Fusions Show a Distinct Profile

To better understand the characteristics of pathogenic fusions in normal tissues, we identified and queried the GTEx cohort for fusion events known to be common in the normal population (polymorphic fusions). These include *KANSL1-ARL17B* and *TFG-GPR128* (Chase et al., 2009; Boettger et al., 2012; Table 1 rows 16–17). These fusions were detected (Figures 1H,I) with high read support in a large number of patients and tissues per patient (Figure 1J), contrasting strongly with the profiles of the *BCR-ABL1* and *SAMD12-EXT1* fusions in healthy individuals.

## Implications for Rare Disease Studies

The identification of putatively pathogenic fusions in a healthy control database has strong implications for the use of a naïve fusion filtering approach that expects no evidence of a pathogenic fusion in a normal expression database. The previously proposed filtering strategies could easily cause the exclusion of important pathogenic fusions, and should be carefully reconsidered. Studies of rare genetic disease typically use non-zero population frequency-based thresholds in variant filtration cascades; a common filter is to remove variants with population frequency >1%. It may be reasonable to adopt a similar threshold for fusion analysis. In our study, the *BCR-ABL1* was detected in approximately 4% of the 500+ GTEx individuals profiled, albeit in a minority of tissues and with low read-support. If each of the 8000+ tissue samples is considered independently, only ∼0.25% of the independent samples profiled contained evidence of *BCR-ABL1* fusions. Thus, using a 1% population frequency filter for fusions occurring in GTEx tissue samples could be a reasonable strategy.

Read-support is another metric which could be considered in a filter strategy. It is possible that fusion transcripts with low read support could be tagged and removed from a normal tissue database to prevent filtering of pathogenic fusions from patient sample analyses. Based upon the data reported here, tagging fusions with two or fewer reads would remove most instances of observed pathogenic fusions from the normal tissue database. This approach was used successfully in our previous study (Oliver et al., 2019b). Arguably, however, such depth-based filtering mechanisms may not be appropriate in all circumstances for several reasons. First, read-support will scale with read-depth and as such needs to be normalized to the study samples used. Second, filtering should not be used in the disease-affected patient samples, as often the affected tissue (e.g., brain or nervous tissue) is inaccessible and surrogate tissue sources such as whole blood are utilized. This may result in low-level evidence of circulating fusion transcripts originating from another tissue or tissues, and/or arising from a mosaic event. In fact, the validated *SAMD12-EXT1* pathogenic fusion was detected with moderate support (17 reads) in patient whole blood in our prior study and was later verified to originate from a mosaic deletion event. Consequently, use of read-support should be considered as a quality control annotation that has been properly parameterized to the datasets under investigation, and not applied as a generic filter threshold.

Finally, using the observed number of tissues a fusion occurs in as a filtering threshold will be problematic. While the suspected pathogenic events in this study were observed in a small number of tissues per healthy individual (Figure 1J), the polymorphic fusions *KANSL1-ARL17B* and *TFG-GPR128* varied widely in the number of tissues in which they were detected (Figures 1H,I). Furthermore, for most clinical studies, RNA data is unlikely to be available from multiple tissues per individual and when it is, incomplete tissue detectability of a fusion may be a characteristic worthy of investigation. As such, this observed characteristic is not a viable filtering metric in isolation, although in combination with read-support and observed population frequencies it may be biologically informative.

Ultimately no single filtering strategy will be suitable for all applications but it is our hope that the considerations raised here empower researchers to make informed decisions about suitable strategies for their own applications.

## Proposed Origins of Pathogenic Events in Normal Tissues

The question of why putatively pathogenic fusions are detected in presumed normal tissue databases is an intriguing one. In the absence of large-scale validation efforts conducted upon the GTEx samples, we are left to theorize possible explanations. Undoubtedly a subset of the community will point to such findings as erroneous or spurious, ultimately classifying these events in the category of “false-positives.” Bioinformatics artifacts are common due to sequence homology, promiscuous alignments or artifacts of gene annotation. Laboratory-based artifacts arising from various components of sample processing and sequencing protocols are similarly infamous. It is for these very reasons that fusion detection algorithms have traditionally required rigorous training on biological or synthetic data sets. In the authors’ opinion, however, numerous facts point toward an alternative explanation. All fused sequence candidates were aligned to the human genome with BLAST and confirmed not to be promiscuous in their genomic alignments, nor share obvious sequence homology. All fusions considered here represent events occurring at precise exon–exon boundaries of two distinct genes. A conservative calculation based on Ensembl transcripts (mean exon length 330 bases) suggests a 3.7e-5 probability that two randomly selected bases occur at exon boundaries. As such, the likelihood that one of these observed fusion candidate events formed though an artifactual *in vitro* or *in silico* processes and not through normal splicing is exceedingly low. What seems more likely in our opinion is that the fused species arise *in vivo*, resulting from the aberrant DNA breakage and repair, and subsequent transcription and splicing. It is widely acknowledged that DNA undergoes constant mutation, breakage and repair, and that certain genomic regions are more susceptible to this due to nucleic proximity or other factors. This combined with genetic mosaicism may explain the presence of pathogenic mutations in a subset of the body’s cells and tissues. Known pathogenic fusion events occurring at low numbers and in select tissues may commonly occur and be rapidly repaired at the genomic level. However, a fraction may escape this and give rise to subclonal cell populations that ultimately remain benign due to an unsuitable tissue environment, or immune detection and clearance. Finally, such subclonal events may be precursors of true neoplastic disease if the body’s defense and repair mechanisms are escaped and local physiological conditions become suitable for proliferative growth. (Whether in fact the observation of such events in healthy, living individuals might indicate a need for clinical follow-up is another question that will require further evidence to answer). Alternatively, mosaic events occurring earlier in development might be more widely detectable but ultimately remain benign based on an insufficiency of affected cells or lack of effect in a given tissue-type. Independently or in unison, these mechanisms could create the observed landscape of detectable pathogenic events and explain the very different detectability profiles observed for polymorphic or potentially pathogenic fusions.

The possibility of sample to sample cross-contamination should also not be discounted. GTEx leukemia cell lines for example might arguably have the potential to contaminate other samples being processed in parallel. However, this would not explain the *SAMD12-EXT1* fusion as it is not known to occur with high frequency in any tissue or cell type profiled by GTEx. Notably we are not the first to have suggested the presence of pathogenic fusions in normal tissues. A follow-up literature review unearthed prior reports of three known pathogenic fusions being detected in normal tissues prior to the era of large-scale sequencing (Fears et al., 1996; Maes et al., 2001), although we were unable to find any evidence of these events occurring within the GTEx data. Ultimately confirmation of the true nature of such events and the absolute measure of their ubiquity will require further study by the scientific community. The authors hope that the dissemination of our observations to the wider field will both inform efforts pertaining to the discovery of pathogenic fusions and inspire an increase in the basic research required to more wholly understand the observation of such events in normal tissues. In a relatively short time period, pathogenic fusion transcripts have progressed from being viewed as hematological cancer specific, to solid tumor ubiquitous, to diagnostic of rare inherited disease and now potentially to being background components of healthy individual’s cells. The question of how or if their relevance continues to increase remains open.

## Data Availability Statement

GTEx data used for the analyses described in this article were obtained from dbGaP accession 280 number phs000424.v7.p2.

## Ethics Statement

The studies involving human participants were reviewed and approved by the Mayo Clinic Institutional Review Board. Written informed consent to participate in this study was provided by the participants’ legal guardian/next of kin.

## Author Contributions

GO performed the data analysis and interpretation, and conceived and wrote the manuscript. GJ performed the data analysis, generated figures, and reviewed the manuscript. EK helped to conceive the study and reviewed the manuscript.

## Conflict of Interest

The authors declare that the research was conducted in the absence of any commercial or financial relationships that could be construed as a potential conflict of interest.
